# The Role of the Disulfide Bridge in the Copper (II) Binding by the Cyclic His_4_-Peptide

**DOI:** 10.3390/ijms22126458

**Published:** 2021-06-16

**Authors:** Aleksandra Pieniężna, Weronika Witak, Aneta Szymańska, Justyna Brasuń

**Affiliations:** 1 Department of Inorganic Chemistry, Wroclaw Medical University, Borowska 211A, 50-552 Wrocław, Poland; aleksandra.pieniezna@umed.wroc.pl (A.P.); weronika.witak@umed.wroc.pl (W.W.); 2 Department of Biomedical Chemistry, Faculty of Chemistry, University of Gdańsk, Wita Stwosza 63, 80-308 Gdańsk, Poland; aneta.szymanska@ug.edu.pl

**Keywords:** cyclopeptides, coordination, copper (II) ions, proline, disulfide bridge, potentiometric measurements, spectroscopy

## Abstract

In this paper, we present studies on the influence of the disulfide bridge on the copper (II) ions’ binding abilities by the cyclic His_4_-peptide. The studied ligand HKHPHRHC_-S-S-_C consists of nine amino acids. The cyclic structure was obtained through a disulfide bridge between two cysteinyl groups. Moreover, this peptide is characterized by the presence of four His residues in the sequence, which makes it an interesting ligand for transition metal ions. The potentiometric and spectroscopic (UV-Vis spectroscopy and circular dichroism spectroscopy (CD)) studies were carried out in various molar ligand to metal ratios: 2:1, 1:1, and 1:2, in the pH range of 2.5–11 at 25 °C. The results showed that the cyclic His_4_-peptide promotes dinuclear complexes in each of these systems and forms the final dinuclear species with the {N_Im_, 3N^-^_amide_}{N_Im_, 3N^-^_amide_} coordination mode. The obtained data shows that cyclization by the formation of the disulfide bond has an impact on the peptide chain flexibility and appearance of additional potential donors for metal ions and influences the copper (II) ions’ coordination.

## 1. Introduction

Cyclopeptides play an important role in studies of metal ion coordination. The formation of the cyclic structure can be obtained by the formation of peptide bond between the N-terminal amino group of the peptide and the carboxylic C-terminus. As a result of this process, the amino group loses its metal coordination properties. Only the nitrogen atoms from peptide bonds [1,2,3,4] and from the peptide side chain groups (e.g., the imidazole ring of the histidine residue) as well as oxygen from the carboxylic group of aspartic acid [1,2,3] are available as donor atoms for metal coordination in the cyclic peptide molecule.

The other way to obtain the cyclic motif in the peptide structure is cyclisation by the formation of the disulfide bridge, which is found, for example, in peptide hormones such as vasopressin or oxytocin and influences their metal ion coordination propensities [5,6]. Vasopressin and its natural or synthetic analogues form complexes with the same stoichiometry as, for example, tetraGly, but the presence of the cyclic structure influences the sterical arrangement of the peptide bonds and facilitates the involvement of amide nitrogens, which makes these peptides more effective in Cu(II) binding in relation to theirs linear analogues. The studies performed by us before for the peptides with the c(HKHPHKHP) [7] and c(HKHGPGHKHGPG) [8] sequences (CM and CP peptides, respectively) showed that, in equimolar conditions, they form the same complexes as a peptide with two Gly residues instead of two Pro residues; however, the presence of Pro facilitates the involvement of a third amide donor. Their binding abilities are notably changed in the system with a double excess of Cu(II) ions. In acidic conditions, it forms only mononuclear complexes, but above pH 6, only binuclear complexes exist. At the physiological range of pH, the binuclear species with both Cu(II) ions bound by four nitrogen donors are present, however, one Cu(II) is coordinated by two N_Im_ and two N_amide_ donors, whilst in the coordination sphere of the second Cu(II) one imidazole and three amide donors are present [7,8]. Despite the growing knowledge of cyclopeptides, the question about the more precise role of the disulfide bridge in their structure and its effect on the coordination of metal ions is still unresolved.

In this paper, we present the results of research on a HKHPHRHC_-S-S-_C (CMS) ligand containing in its structure a disulfide bridge and Pro residue introduced to assess their common effect on the coordination properties regarding the binding abilities of cyclopeptide toward copper (II) ions. The proline is a special amino acid residue that notably influences the coordination abilities of peptides and, due to its cyclic structure, effectively prevents the coordination of subsequent amide nitrogens [9,10]. The analysis of the obtained results in relation to the previously studied peptides CM and CP allows for the role of the disulfide bridge in copper (II) binding by His4-cyclopeptides to be characterized.

## 2. Results and Discussion

The acid–base properties of the ligand were determined at the first stage of research. An analysis of potentiometric results (Table 1) showed that the researched peptide has six deprotonation constants. The protons of the imidazole rings of the histidyl residues dissociate in the pH range of 5–7. Further constants are characteristic of the dissociation of the proton from the lysine side chain amino group (9.71) and the dissociation of the proton from the arginine residue (11.12).

**Figure 1:**
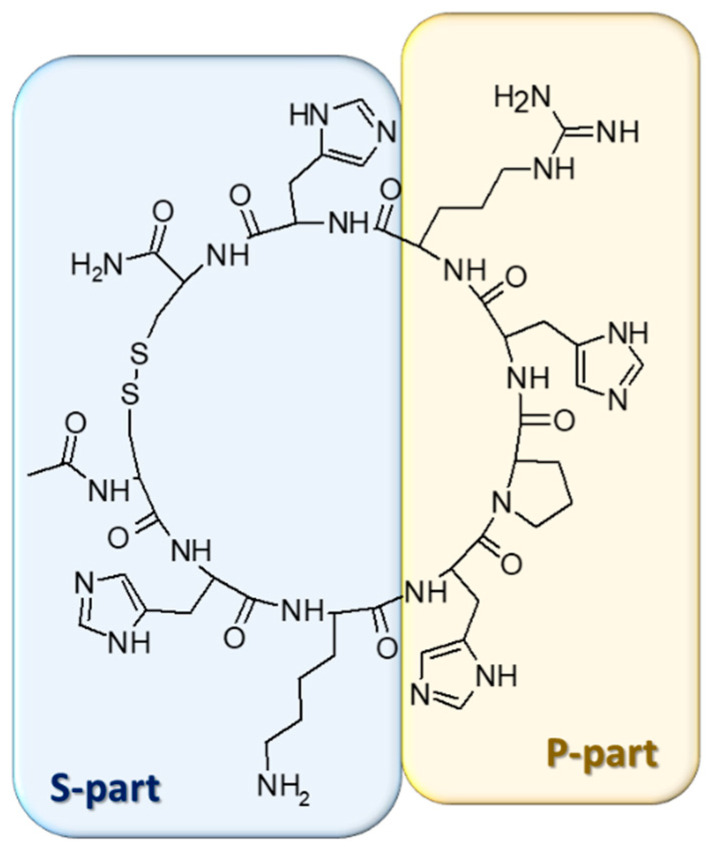
Structure of ligand CMS detailing the two parts: the P-containing proline residue and the S-part containing disulfide bridge.

The presence of one Pro residue and one S-S bond in the peptide cycle creates two different domains capable of Cu(II) binding that differ in the flexibility of the ligand unit of the peptide chain (Figure 1).

**Figure 2:**
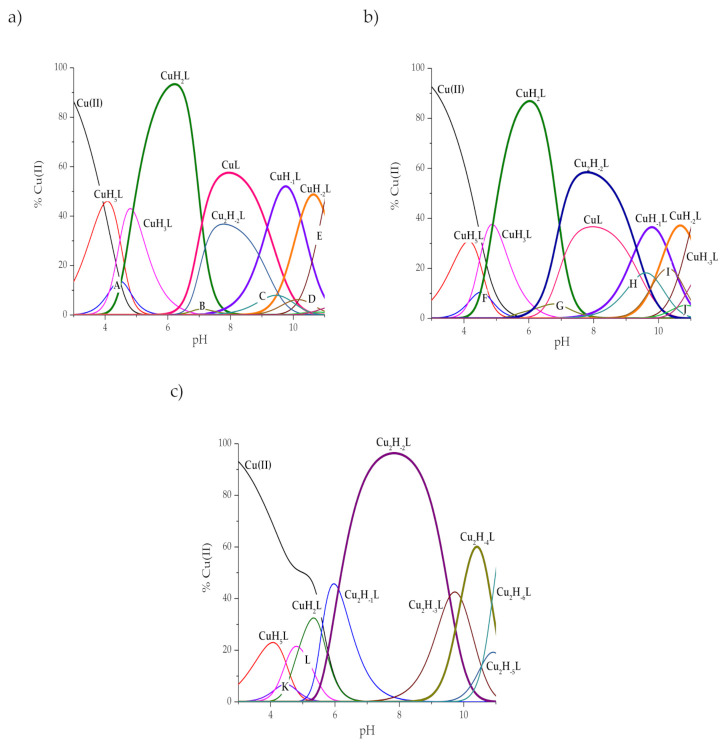
The species distribution curves for the system of CMS with Cu(II) ions. The ligand to metal ratios of (**a**) 2:1, (**b**) 1:1, and (**c**) 1:2 are shown. The minor species are marked as letters to enhance clarity: A: CuH_4_L; B: Cu_2_H_−1_L; C: Cu_2_H_-3_L; D: Cu_2_H_−4_L; E: CuH_−3_L; F: CuH_4_L; G: Cu_2_H_−1_L; H: Cu_2_H_-3_L; I: Cu_2_H_-4_L; J: Cu_2_H_-5_L; K: CuH_4_L; L: CuH_3_L.

The analysis of the potentiometric results shows that the investigated peptide forms series of mono- and dinuclear complexes. Figure 2 presents the formation of the complexes in dependence on pH at different ligand to metal molar ratios. The respective stability constants are collected in Table 1. The comparison of the coordination profiles between different ligand to metal molar ratios shows that systems with 2L:1Cu(II) and 1L:1Cu(II) are similar. The main difference is observed at pH 8, where, in the system with excess of the ligand, the CuL species dominates, whilst in the second system the dinuclear complex, Cu_2_LH_-2_ represents the main coordination mode. Owing to this fact, these two systems will be discussed together.

Below pH 6, four complexes exist: CuH_5_L, CuH_4_L, CuH_3_L and CuH_2_L. Due to the low concentration and coexistence of different copper (II) complexes, it was difficult to obtain the spectroscopic parameters for the first three complexes. However, the values of the corrected stability constants: *logβ^∗^_(CuH5L)_* = 3.92, *logβ^∗^_(CuH4L)_* = 4.77, and *logβ^∗^*_(CuH3L)_ = 6.72, where *logβ^∗^* *= logβ_CuHnL_-logβ_HnL_*, are comparable to *logβ^∗^* complexes with one, two, and three imidazole donors, respectively in the coordination sphere of the Cu(II) ion [7,11,12,13,14]. The next appearing species, achieving its highest concentration at pH 6, is CuH_2_L. The value of logβ*_(CuH2L)_ = 8.61 is comparable to logβ*_(CuH2L)_ = 8.46, calculated for the 4N_Im_-type complex formed by the complex previously analyzed by us, the CM peptide with the c(PHKHPHKH) sequence [7]. The formation of this complex notably influences the spectral abilities of the system.

**Figure 3:**
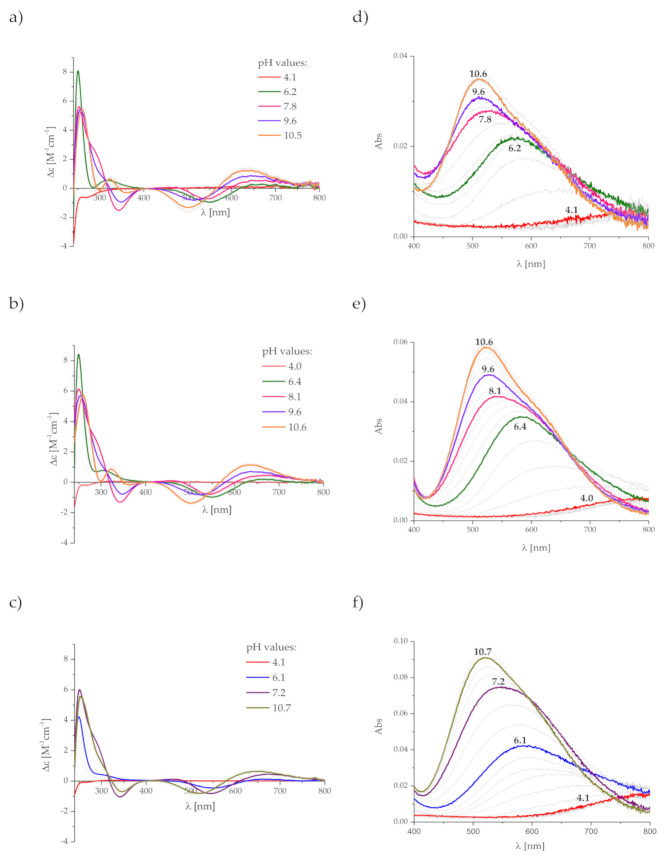
The circular dichroism spectra for solutions with the following ligand to metal molar ratios: (**a**) 2:1 (**b**) 1:1, and (**c**) 1:2, and the UV-Vis spectra for solutions with following ligand to metal molar ratios: (**d**) 2:1 (**e**) 1:1, and (**f**) 1:2. Measurements were recorded at pH ranges of 2.5–11.0.

The appearance of two positive CT_L__→M_ bands at 260 nm and 320 nm in the circular dichroism spectrum supports the involvement of the imidazole donors in metal binding (Figure 3a,b) [12,15,16]. Moreover, the location of λ_max_ d-d in absorption spectrum at 575 nm is in agreement with the theoretical λ_max_ = 585 nm (Figure 3d,e) calculated for the Cu(II) complex with four imidazole donors in the plane [17].

With the pH increase, the next two complexes, CuL and Cu_2_H_-2_L, appear with the highest concentrations at pH 8. In the system with the molar ratio nL:nCu(II) = 2:1, the CuL complex and Cu_2_LH_-2_ are dominant, with the concentration of 60% and 40%, respectively, whilst in the system with the nL:nCu(II) = 1:1 molar ratio, the situation is opposite. The formation of the CuL is related to dissociation of two protons from CuH_2_L and formation of the complex with the {2N_Im_, 2N^-^_amide_} coordination mode. The situation is more complicated in the case of dinuclear species, where two possibilities for the coordination of copper (II) ions can be proposed: i/ {2N_Im_, 2N^-^_amide_}{2N_Im_, 2N^-^_amide_}, ii/ {N_Im_, 3N^-^_amide_}{3N_Im_, N^-^_amide_}. Nevertheless, the spectroscopic parameters obtained at pH 8 for both systems (i.e., the blue shift of the of the λ_max_ for d-d transitions (≈30 nm)) support the possible replacement of the imidazole nitrogens by amide donors, which are stronger ligands for Cu(II) ion. Nevertheless, the formed species is still 4N-type. This assumption is supported by the appearance of a new negative CT transition at ≈342 nm [12,15,16]. The presence of two positive transitions at ≈249 nm and ≈288 nm in the CD spectrum confirms that the imidazole donors are still involved in the metal binding [12,16].

Above pH 9, the next mono- and dinuclear complexes appear in the discussed system; however, the mononuclear species (CuLH_−1_, CuLH_−2_, and CuLH_−3_) are dominant, especially in the system with the two-fold excess of the ligand. The formation of these complexes can be attributed to the dissociation of three protons: two from the Lys and Arg side chains and one from the third amide, and the formation of the species with the 1 × N_Im_, 3 × N^−^_amide_ donors in the coordination sphere of Cu(II) ion.

The analysis of the system with a two-fold excess of Cu(II) (Figure 2c) shows that the acidic conditions promote the formation of the mononuclear complexes, whilst above pH 6, only dinuclear species exist in the system, as was observed in previous studies [7,8]. It is difficult to unambiguously define the coordination manner, but the location of the d-d- band at 518 nm supports the presence of the 4N-type of copper (II) complexes [7,12,15], while CT bands at 250 nm and 340 nm confirm the involvement of the imidazole and amide donors in the metal ion coordination [7,12,16].

**Scheme 1:**
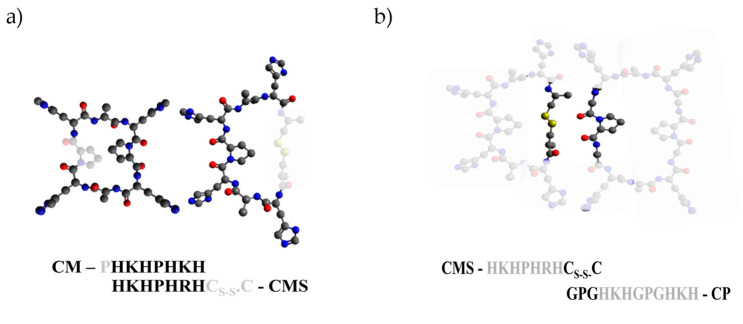
The differences and similarities in the structures of peptides (**a**) CM and CMS, (**b**) CMS and CP. CM: c(HKHPHKHP); CP: c(HKHGPGHKHGPG).

**Figure 4:**
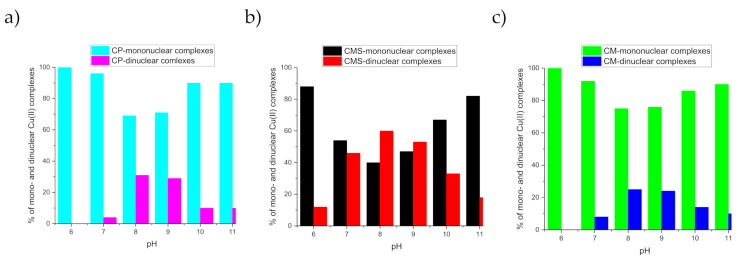
A comparison of the ability to form mono- and dinuclear complexes by peptides with Scheme 1. for (**a**) CP, (**b**) CMS, and (**c**) CM.

The aim of this work was to characterize the influence the -S-S- bridge on the copper (II) binding by the model His_4_-cyclopeptide. The replacement of the one Pro residue by the -S-S- bond makes the studied CMS peptide the chimera of the CM and CP ligands. The replacement of one of the Pro resides from CM peptide by two Cys enables another mode of peptide cyclization (the formation of the disulfide bridge) (Scheme 1a). The His4 metal binding sequence is the same in all compounds (-HKHPHXH-, where X = Lys or Arg). The comparison of the binding abilities between CMS, CM, and CP provides interesting observations. First of all, CMS, due to its more flexible chain, promotes the formation of the dinuclear Cu(II) complexes in contrast to the CM and CP, even in the system with an excess of the ligand (Figure 4a,b).

The observed results support the hypothesis that the formation of dinuclear complexes is promoted by the flexibility of the peptide chain rather than by the number of the amino acid residues in the peptide cycle.

For a more detailed analysis, a comparison of the stability constants independent of the protonation states of basic side chain groups of both Lys (CM, CP) or Lys and Arg (CMS) was performed (Table 2).

The CMS was observed to form the same main complexes as CM and CP. The comparison of the stability constants of the mononuclear complexes shows similar stabilities of complexes, with only imidazole chromophores in the coordination sphere formed by CMS and CM. This changes when the amide donors start to be involved in coordination, which is also observed for dinuclear complexes (Table 2).

The presence of the disulfide bond also influences the copper (II) ions’ coordination mode, however, an interaction between S-donors and Cu(II) was not observed. The CM peptide, first from the His_4_-cyclopeptide-investigated ligands, forms the final dinuclear species with the {N_Im_, 3N^−^_amide_}{2N_Im_, 2N^−^_amide_} coordination mode [7]. The increase of the peptide ring by the insertion of the additional amino acid residues (4xGly) does not promote the involvement of the third amide donor in the final di-copper (II) species (CP forms the final complex with the {2N_Im_, 2N^−^_amide_}{2N_Im_, 2N^−^_amide_} binding mode [8]). In contrast to CM and CP, CMS, with a more flexible peptide cycle, forms the final Cu(II)/Cu(II) complex with the {N_Im_, 3N^−^_amide_}{N_Im_, 3N^−a^_amide_} binding mode.

## 3. Materials and Methods

### 3.1. The Synthesis of the Ligand

CMS peptide was obtained by means of microwave-assisted solid phase peptide synthesis using Liberty Blue peptide synthesizer (CEM, USA, Matthews, NC). TentaGel R RAM resin was used, and the process was carried out using standard instrument settings, recommended by the manufacturer for Fmoc-amino acids chemistry. After, the synthesis peptide was removed from the solid support together with the side chain protecting groups using trifluoroacetic acid (TFA)-based cleavage cocktail (TFA:H_2_O:triizopropylsilane:phenol, 88:5:2:5, *v*/*v*) under an argon atmosphere, precipitated with diethyl ether and lyophilized. Crude linear peptide was cyclized by air-oxidation of the sulfhydryl group. Peptide was dissolved in 1.5 L of a water:MeOH mixture (1:9, *v*/*v*) and left in an open flask with stirring. The process was monitored using RP-HPLC on Kinetex column (2.6 µm, C8, 100A, 100 × 2.1 mm) operated by Nexera-i chromatography system (Shimadzu, Japan, Kioto,). After 48 h, cyclization was complete.

Crude cyclic peptide was recovered by evaporation of the solvents and lyophilization. Purification was accomplished using RP-HPLC chromatography on a Luna (5 µm, C8(2), 100A, 250 × 21.2 mm) LC-column using a linear gradient 2–50% B over 90 min (where A—0.1% TFA in water, B—80% CH3CN in A). Pure fractions (purity > 98%) were collected, evaporated, and lyophilized to yield 41.5 mg of final product (35%). Peptide identity was confirmed by mass spectrometry (LC-MS ESI-IT-TOF, Shimadzu, Japan, Kioto).

### 3.2. Potentiometric Measurements

The pH-metric titrations were carried out using a Metrohm (Switzerland, Herisau) pH-meter system with a semimicro combined electrode calibrated in hydrogen ion concentration using HCl at 25 °C [18] in a constant ionic strength of 0.1 M KCl. The ligand concentration was 1 × 10^−3^ M and ligand to Cu(II) metal ion molar ratios were 1:1, 2:1, and 1:2. KOH was added using a 0.250-mL micrometer syringe. The concentration of KOH was 0.1 M. Measurements were carried out in the 2.5–11 pH range. The SUPERQUAD 5.20 software and HYPERQUAD 2008 computer programs were used to calculate the protonation constants of the ligands and the stability constants (*β*) of the copper complexes using Equations (1) and Equation (2) [19,20].

(1) $$aM+bH+cL⇋M_{a}H_{b}L_{c}$$

(2) $$\beta_{abc}=\frac{\left[M_{a}H_{b}L_{c}\right]}{{\left[M\right]}^{a}{\left[H\right]}^{b}{\left[L\right]}^{c}}$$

### 3.3. Spectroscopic Studies

Absorption spectra of the copper (II) complexes were recorded at 25 °C on a Varian Cary 50 Bio spectrophotometer (Varian Inc., USA, Palo Alto, California) in 1-cm path length quartz cells. All UV-Vis spectra were collected in the 350–900 nm and 2.5–11 pH range. Circular dichroism (CD) spectra were recorded on a Jasco J-1500 spectrophotometer (Jasco, Japan, Tokyo) in the 240–800 nm range using 1-cm cuvettes. The same concentration, ionic strength, and molar ratios were used for both spectroscopic and potentiometric studies.

## 4. Conclusions

The influence of the disulfide bridge on the binding abilities of copper (II) ions by the cyclic His_4_-peptide was presented in this paper. CMS has six deprotonation constants and forms series of mono- and dinuclear complexes at metal to ligand ratios of 2:1 and 1:1, but also 1:2. Systems with 2L:1Cu(II) and 1L:1Cu(II) are similar. At pH 6, the CuH_2_L species were observed to achieve the highest concentration. The appearance of this complex notably influences the spectral abilities of the system. The analysis of the system with a two-fold excess of Cu(II) shows that the formation of the mononuclear complexes is promoted in acidic conditions and above pH 6 only dinuclear complexes exist. Studies show that the presence of the disulfide bond influences the coordination of the copper (II) ions. Furthermore, a comparison with previously investigated peptides, CM and CP, showed the influence of the flexibility of the peptide chain on the formation dinuclear complexes.

## Figures and Tables

**Table 1:** The potentiometric data for CMS (HKHPHRHC_-S-S-_C) ligand at 25 °C and I = 0.1 M. The ligand concentration was 1 × 10^−3^ M.

CMS—HKHPHRHC_-S-S-_C							
	HL	H_2_L	H_3_L		H_4_L	H_5_L	H_6_L
*logβ*	11.12 ± 0.01	20.83 ± 0.01	27.61 ± 0.02		33.69 ± 0.02	39.36 ± 0.02	44.34 ± 0.02
*logK*	11.12	9.71	6.78		6.08	5.67	4.98
Mononuclear complexes				Dinuclear complexes			
		*logβ*				*logβ*	
Species				Species			
CuH_5_L		43.28 ± 0.04		Cu_2_H_-1_L		15.58 ± 0.03	
CuH_4_L		38.46 ± 0.10		Cu_2_H-_2_L		9.47 ± 0.02	
CuH_3_L		34.33 ± 0.03		Cu_2_H_-3_L		−0.09 ± 0.05	
CuH_2_L		29.44 ± 0.02		Cu_2_H_-4_L		−10.01 ± 0.04	
CuL		15.26 ± 0.04		Cu_2_H_-5_L		−21.18 ± 0.11	
CuH_-1_L		6.09 ± 0.05		Cu_2_H_-6_L		−31.75 ± 0.04	
CuH_-2_L		−4.15 ± 0.04					
CuH_-3_L		−15.10 ± 0.04					
		*logK*				*logK*	
*logβ_CuH5L_—logβ_CuH4L_*		4.82		*logβ_Cu2H-1L_—logβ_Cu2H-2L_*		6.11	
*logβ_CuH4L_—logβ_CuH3L_*		4.13		*logβ_Cu2H-2L_—logβ_Cu2H-3L_*		9.56	
*logβ_CuH3L_—logβ_CuH2L_*		4.89		*logβ_Cu2H-3L_—logβ_Cu2H-4L_*		9.92	
*logβ_CuH2L_—logβ_CuL_*		14.18		*logβ_Cu2H-4L_—logβ_Cu2H-5L_*		11.17	
*logβ_CuL_—logβ_CuH-1L_*		9.17		*logβ_Cu2H-5L_—logβ_Cu2H-6L_*		10.57	
*logβ_CuH-1L_—logβ_CuH-2L_*		10.24					
*logβ_CuH-2L_—logβ_CuH-3L_*		10.95					

**Table 2:** The comparison of the corrected stability constants between CMS, CM, and CP for the complexes with the same binding manner.

The Binding Manner	CMS	CM [7]	CP [8]
	Mononuclear complexes		
{2N_Im_} {3N_Im_} {4N_Im_} {2N_Im_,2N^-^_am_} {1N_Im_,3N^-^_am_}	17.63 13.50 8.61 −5.57 −15.10	17.20 - 8.46− 6.34− 15.81	19.02 - 9.81 −5.41 −14.69
	Dinuclear complexes		
{2N_Im_,2N^-^_am_}{3N_Im_,1N^-^_am_} {2N_Im_,2N^-^_am_}{2N_Im_,2N^-^_am_} {1N_Im_,3N^-^_am_}{2N_Im_,3N^-^_am_} {1N_Im_,3N^-^_am_}{1N_Im_,3N^-^_am_}	−5.25 −11.36 −20.92 −31.75	−7.13 −14.23 −22.87 -	−5.64 −11.64 - -

## Data Availability

Not applicable.
